# Older Adults’ Experiences of Behavior Change Support in a Digital Fall Prevention Exercise Program: Qualitative Study Framed by the Self-determination Theory

**DOI:** 10.2196/26235

**Published:** 2021-07-30

**Authors:** Beatrice Pettersson, Rebecka Janols, Maria Wiklund, Lillemor Lundin-Olsson, Marlene Sandlund

**Affiliations:** 1 Physiotherapy, Department of Community Medicine and Rehabilitation Umeå Sweden; 2 Occupational Therapy Department of Community Medicine and Rehabilitation Umeå University Umeå Sweden; 3 Department of Computing Science Umeå University Umeå Sweden

**Keywords:** accidental falls, aged, exercise, qualitative research, eHealth, self-management, fall prevention, behavior change, self-determination theory, classification of motivation and behavior change techniques

## Abstract

**Background:**

Exercise is an effective intervention to prevent falls in older adults; however, long-term adherence is often poor. To increase adherence, additional support for behavior change has been advocated. However, consistency in the reporting of interventions using behavior change techniques is lacking. Recently, a classification system has been developed to increase consistency in studies using behavior change techniques within the self-determination theory.

**Objective:**

This study aimed to explore expressions of self-determination among community-dwelling older adults using a self-managed digital fall prevention exercise program comprising behavior change support (the Safe Step program), which was developed in co-creation with intended users.

**Methods:**

The qualitative study design was based on open-ended responses to questionnaires, and individual and focus group interviews. A deductive qualitative content analysis was applied using the classification system of motivation and behavior change techniques as an analytical matrix, followed by an inductive analysis. Twenty-five participants took part in a feasibility study and exercised in their homes with the Safe Step program for 4 months. The exercise program was available on computers, smartphones, and tablets, and was fully self-managed.

**Results:**

In the deductive analysis, expressions of support were demonstrated for all three basic human psychological needs, namely, autonomy, competence, and relatedness. These expressions were related to 11 of the 21 motivation and behavior change techniques in the classification system. The inductive analysis indicated that autonomy (to be in control) was valued and enabled individual adaptations according to different rationales for realizing exercise goals. However, the experience of autonomy was also two-sided and depended on the participants’ competence in exercise and the use of technology. The clarity of the program and exercise videos was seen as key for support in performance and competent choices. Although augmented techniques for social support were requested, support through relatedness was found within the program.

**Conclusions:**

In this study, the Safe Step program supported the establishment of new exercise routines, as well as the three basic human psychological needs, with autonomy and competence being expressed as central in this context. Based on the participants’ experiences, a proposed addition to the classification system used as an analytical matrix has been presented.

**Trial Registration:**

ClinicalTrials.gov NCT02916849; https://clinicaltrials.gov/ct2/show/NCT02916849

## Introduction

### Background

Fall prevention interventions for older adults living in the community have the potential to reduce the physical and psychological distress resulting from a fall [1]. A recent meta-analysis showed that physical exercise, in particular, is an effective intervention for the reduction of fall rates and number of falls [2]. However, fall prevention exercise interventions have failed to produce lasting long-term results owing to a lack of successful behavior change and sustained exercise routines among participants [3,4]. To better understand older adults’ exercise adherence and health behavior, the research community has identified a need to increase the use of behavioral scientific knowledge in the development, use, evaluation, and reporting of interventions [5-7]. However, interventions aimed at supporting increased physical activity and improving adherence to interventions among older adults are rarely underpinned by behavior change theories [5].

There is growing interest in using digital technology to support behavior change, increase access to fall prevention interventions, and promote the self-management of physical activity [8-10]. Older adults have expressed an appreciation for the increased autonomy and flexibility provided by digital programs, which allow them to exercise at a time that suits them in a location of their choosing [11-13]. Moreover, the incorporation of individual and social behavioral change components in digital exercise programs has proven more successful in improving adherence and physical performance among older adults in comparison with those receiving a home exercise booklet [14]. There is still a great need to further investigate the feasibility of self-managed digital exercise interventions for older adults. Notably, a systematic review indicated that techniques that increase self-efficacy and physical activity behavior in a younger population are not as effective in adults aged 60 years or older [15]. Consequently, involving end-users in the design and development of new programs could be a way to increase the adoption of health-related digital interventions among older adults. It is of major importance that the interventions are reported in a structured way and evaluated in terms of which behavior change techniques older adults find supportive and in which context.

The aim of this study was to explore expressions of self-determination among community-dwelling older adults using a self-managed digital exercise program for fall prevention with co-created behavior change support, and to explore a classification system based on self-determination theory (SDT) as an analytical matrix in this self-managed digital context.

### SDT and Classification of Motivation and Behavior Change Techniques

SDT points to the individual’s perceived self-regulation and internalization process as an explanatory factor in behavioral outcomes [16]. Perceived self-regulation is based on the “satisfaction” or “frustration” of the following three basic human psychological needs: *autonomy*, *competence*, and *relatedness* [17]. Autonomy represents the feeling of self-endorsement or volition in one’s behavior. Competence represents a feeling of capability and reflects an ambition to perform and improve skills. Lastly, relatedness represents being a significant part of a social context, which evokes feelings of being connected and valued. Autonomy is central to SDT, as it is through this construct that the other basic psychological needs are actualized. Therefore, the needs determine the quality of motivation, but they are dependent on the level of autonomy [18]. According to SDT, all three basic human psychological needs have to be satisfied in order to achieve self-motivation and experience well-being [16].

It has been concluded that techniques used within SDT-based interventions cannot fully be captured by existing taxonomies [19,20]. Therefore, the classification of motivation and behavior change techniques (MBCTs) was recently developed in an expert consensus by Teixeira et al [20]. The purpose was to increase consistency in the identification and reporting of techniques between studies using SDT in health contexts. The classification comprises 21 MBCTs organized according to the techniques’ support for each psychological need, accompanied by labels, definitions, and function descriptions. An MBCT is defined as “a distinct, observable, and replicable component of an intervention, designed to influence a person’s behavior directly or indirectly by impacting the person’s perceptions of autonomy, relatedness, and/or competence need satisfaction in relation to a particular behavior or group of related behaviors” [20].

## Methods

### Study Setting and Design

This study is part of a larger project with the overarching aim to develop and evaluate self-managed digital fall prevention for older adults. An overview of the whole project and its parts is presented in Multimedia Appendix 1.

Together with older adults, we have co-created behavior change support for a digital self-managed exercise program. The behavior change components were integrated in an application and tested in a feasibility study for 4 months. This qualitative study involved participants using this digital program, and it presents the findings of individual and focus group interviews, as well as open-ended responses to questionnaires, all of which were part of the feasibility study conducted in Umeå, Sweden (ClinicalTrials.gov NCT02916849) between September 2016 and February 2017. The feasibility study compared two home-based self-management exercise programs (a digital program [Safe Step v.1] and a paper booklet) [21]. Participants who enrolled in the study could choose the digital program or the paper booklet. In all, 29 participants chose the digital program. Deductive qualitative content analysis was used to explore the older adults’ experiences of the behavior change support. The classification of MBCTs served as an analytical matrix [20]. Further explorations were made through inductive qualitative content analysis [22]. The Consolidated Criteria for Reporting Qualitative Research (COREQ) [23] were used to guide the reporting.

### Development of the Program and Behavior Change Support

**Figure 1:**
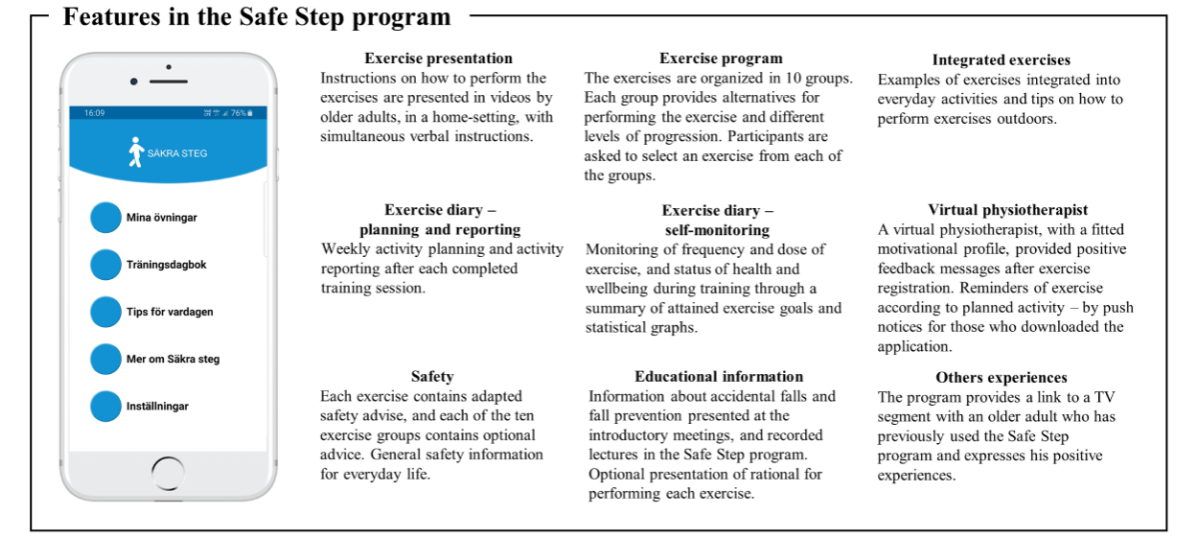
Overview of features in the Safe Step program.

In two steps, the Safe Step program and the behavior change support evaluated in this study were developed by researchers from Umeå University and Luleå Technical University together with older adults, with the majority of external funding coming from government funding bodies. The development of the program was facilitated by the method Participatory and Appreciative Action and Reflection [24]. An overview of features developed in the program is presented in Figure 1. In the first step, a self-management digital exercise program, consisting of, for example, exercise presentation, an exercise program, and integrated exercises, was developed by a multidisciplinary team of researchers in collaboration with older adults [25]. In the second step, through further co-creation, specific behavior change techniques, that is, an exercise diary including planning, reporting, and self-monitoring, as well as a virtual physical therapist, were jointly developed by older adults and researchers with competence in physical therapy and usability.

### The Safe Step Digital Exercise Program

The Safe Step program was available on computers, smartphones, and tablets, and was free of charge. An introduction meeting provided a short introduction to fall prevention and how to navigate and use the digital program. The program was fully self-managed, that is, participants were asked to plan, execute, and evaluate the training independently. As part of the self-management of exercise, the participants were expected to compose their own program of 10 exercises from predetermined groups of exercises with a different focus as follows: lower-limb muscle, balance, and gait/step. The exercises were presented through videos, where older adults performed the exercises in a home setting. All participants were asked to exercise at least 30 minutes, 3 days a week, for 4 months. As part of the feasibility study, participants were offered to participate in a peer-mentoring meeting once a month, which one-third of the participants chose to do.

### Data Collection

Data for descriptive purposes were collected at baseline through a questionnaire containing demographic factors, previous use of technology, and medical history. Data analyzed in this study were collected at two timepoints. Questionnaires were answered 2 months after study start, and focus group interviews and individual interviews were conducted at the end of the 4-month intervention study. An overview of the focus areas for the questions asked on the different data collection occasions is presented in Multimedia Appendix 2. Among the three data collections, 10 participants contributed in one data collection, eight participants contributed in two data collections, and seven participants contributed in all three. In total, 25 participants contributed in the data collection. Their mean exercise time with the program per week was 78 minutes (minimum 11 minutes, maximum 158 minutes).

#### Questionnaires

At 2 months, three participants had withdrawn from the study. The remaining 26 participants received questionnaires, which were sent by mail and contained a postage-paid envelope for the return of the questionnaires. Questions regarding their experiences using the Safe Step program were asked, particularly relating to the individual exercise program, the ease of using of the application, and experiences of the different features, such as planning their weekly exercise and the virtual physical therapist. Each of the questions was accompanied by a text field in which the participants were encouraged to write explanatory comments. Of the 26 returned questionnaires, 18 had comments, which were included as units of analysis in this study. The comments varied from a few words to extensive comments.

#### Focus Group Interviews

At 4 months, two more participants had withdrawn from the study. A purposely selected sample of 14 participants was sent an invitation to participate in focus group interviews. We aimed for a representation of both men and women who had used the program on computers, smartphones, and tablets, as well as variation of exercise frequency. The participants could choose between two different dates to attend the focus group interviews, which were held in a conference room at the university. One woman who signed up for focus group 1 and one man who signed up for focus group 2 withdrew before the interviews began, which resulted in a more unequal distribution of gender than intended. Two moderators conducted the interviews (BP, a physical therapist; RJ, a usability expert), both with previous experience conducting focus group interviews. The interviews were 92 and 87 minutes in length and were facilitated by a focus group guide with open-ended questions (Multimedia Appendix 2). The questions focused on the participants’ experiences of the behavior change techniques developed in the second part of the program development, that is, the exercise diary, the virtual physical therapist, feedback messages and reminders, the approach to make a weekly plan for the exercises, and self-monitoring features. The initiation question concerned the experience of exercising with a program in a digital format.

#### Individual Interviews

A purposive selection of 17 participants, out of the 24 who had completed the digital exercise program, were asked to participate in individual semistructured interviews by a member of the research team at the postassessment of the feasibility study. In order to obtain a varied selection of participants, selection was made based on gender, place of recruitment, and exercise adherence. One of the interviews was conducted with a married couple who both used the digital program. According to the participants’ preference, the interviews were held at the university, at home, or at a library. An interview guide with open-ended questions facilitated the interviews (Multimedia Appendix 2). The questions were related to the participants’ experience of self-managing their exercise and included those regarding the composition of the individual program, perceived effects of the exercise, strategies for and feelings of safety while performing the exercise, strategies and support for maintenance of the exercise, structure and content of the program, use of the program (when, where, and how), and recording in the exercise diary. Follow-up questions were asked, and the participants were encouraged to give concrete examples of their experiences. The interviews were performed by a physical therapist with extensive experience conducting interviews or by one of two physical therapy students with experience conducting interviews. These interviews have previously been analyzed in a study, but with another aim and with both intervention groups included [11]. Background characteristics of the participants are presented in Table 1.

**Table 1:** Participant characteristics.

Characteristic			Questionnaires (n=18)		Focus group 1 (n=6)		Focus group 2 (n=6)		Individual interviews (n=17)
Age (years), mean (min-max)			76.5 (71-91)		76 (72-79)		74 (71-79)		76 (71-91)
Women, n (%)			9 (50%)		1 (17%)		4 (67%)		10 (59%)
Access to a smartphone/tablet, n (%)			14 (78%)		3 (50%)		4 (67%)		14 (82%)
Access to a computer, n (%)			15 (83%)		6 (100%)		5 (83%)		14 (82%)
**Household situation, n (%)**									
	Living together	13 (72%)		3 (50%)		4 (67%)		12 (71%)	
	Living alone	5 (28%)		3 (50%)		2 (33%)		5 (29%)	

### Data Analysis

All interviews were audio-recorded and transcribed verbatim. The data analysis was performed using qualitative content analysis in two stages (deductively and then inductively) [26]. Qualitative content analysis is suitable for exploring variations within data as it highlights differences and similarities [22,27]. The deductive (theory-driven) approach enables exploration of previous knowledge in a different context [26], whereas the inductive (data-driven) approach generates new knowledge by searching for patterns in the data and moving toward a more abstract level of understanding [28].

The classification of MBCTs was used as a categorization matrix in the deductive analysis [20] with the 21 MBCTs as predetermined categories. To capture experiences of self-determination not addressed by MBCTs, the three basic psychological needs tied to SDT [16] were used as main categories in the categorization matrix. Initially, the first author (BP) performed a read through to get a sense for the entirety of the material. Units relevant to the aim of this study were selected and condensed, and representative codes were created. The codes were simultaneously sorted in the categorization matrix. Following the deductive stage, an inductive stage was initiated to compare the codes for similarities and differences and form subcategories within each category of the classification of MBCTs. Data analysis was performed using the software MAXQDA 2020 (VERBI Software). In the context of this study, 10 MBCTs were found to be nonrepresentative. Moreover, codes perceived as corresponding to psychological needs, but not exemplified in the classification system, served as a basis for an additional competence-based category. The analysis was continuously discussed and agreed upon by the authors BP, MW, LLO, and MS. To ensure trustworthiness, these authors also deductively analyzed one individual interview and discussed their interpretations [22]. The emergent results were discussed with the second author (RJ) who was also part of data collection.

## Results

### Overview

Participants’ expressions of self-determination showed that the Safe Step digital exercise program was perceived as a supportive structure that guided their training, providing a feeling of freedom of choice and ownership over their courses of actions. With increased confidence in their actions, the participants were able to find their own meaningful and motivating ways to work with the program. Still, the digital exercise program could entail both freedom and restrictions due to differences in confidence in managing the program and varying needs for social connectedness.

**Figure 2:**
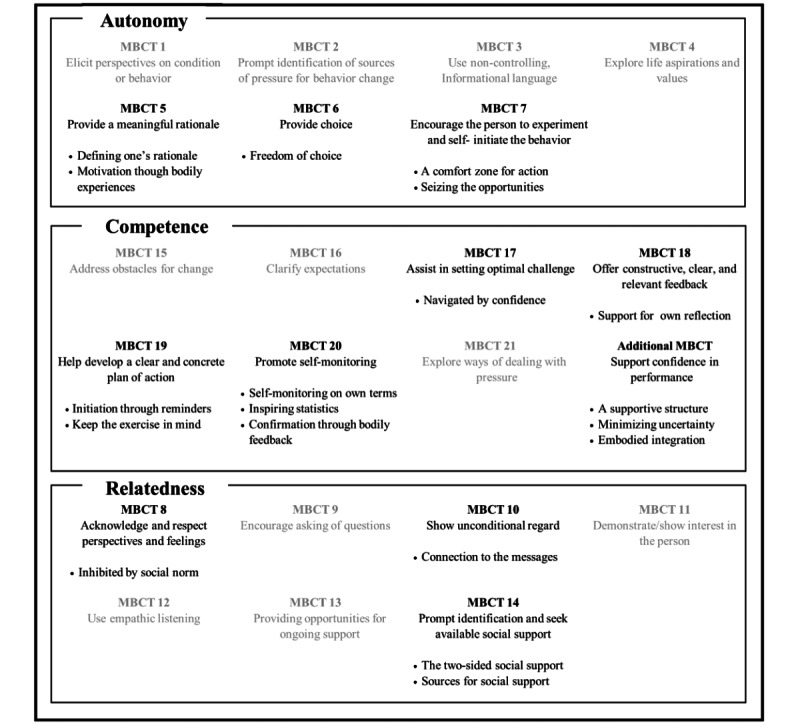
Overview of the motivation and behavior change techniques (MBCTs) according to the classification of MBCTs, where MBCTs found nonrepresentative of our study are presented in grey. The basic human psychological needs are presented in the order in which the participants expressed the most support. The bullets represent subcategories.

As a result of the deductive analysis, we found expressions for all three psychological needs in the SDT [16] (the main categories). Expressions of autonomy and competence were found to be more central than relatedness. Furthermore, the expressions corresponded to 11 of the 21 MBCTs (the categories), and an additional competence-based category was created. In turn, the inductive analysis generated 19 subcategories within these categories. The results are presented in an overview in Figure 2.

### Autonomy Supportive Techniques

The autonomy supportive categories represented the core of the participants’ expressions of support for changes in exercise behavior. Choices regarding exercises and exercise routines were a prerequisite for feeling ownership over their actions and meaningfulness in their performance. Thus, “satisfaction” of autonomy was primarily conveyed.

#### Provide a Meaningful Rationale (MBCT 5)

The identification of a meaningful rationale for performing the exercises was captured in the subcategories “Defining one’s rationale” and “Motivation through bodily experiences.”

The digital program either supported existing rationales for doing the exercises or helped participants develop new rationales. The motivation for doing the exercises was often expressed in terms of long-term goals, such as strengthening leg muscles or being able to dress while standing. Participants also reported a fear of deteriorated psychological and physical health, and the exercises were perceived as a tool to counteract this type of decline and maintain improvements.

This has been good for me, so I cannot suddenly stop. I might get my problems back then.

Woman 1; individual interview

Participants also conveyed an awareness of the economic burden of falls on society. This knowledge had been gained in the introduction of the study; therefore, this had also become a meaningful rationale for doing the exercises. Another rationale and further motivation involved experiences of bodily improvements, which confirmed the effectiveness of the exercise performed. These meaningful improvements of strength and balance were noted not only in everyday activities, such as walking up the stairs, but also when performing the Safe Step exercises. Performing the exercises acted as an eye opener for their issues with balance, which served as a motivator to continue doing the exercise. One participant made the following comment:

When it gets easier to do, it becomes fun, and then enjoyable.

Woman 2; individual interview

#### Provide Choice (MBCT 6)

Different aspects of ownership over the course of action through choices were highlighted in the subcategory “Freedom of choice.”

Freedom of choice was obtained by being able to choose both a time and place for exercise, what device the program would be used on, and which program features to use. The program was utilized according to participants’ own wishes, which created a feeling of ownership over their choices of actions. The routines participants developed were not seen as sustainable if they were not able to find the right arrangement for themselves, put as follows:

There has to be choices for all the different ways of thinking.

woman; focus group interview 2

The wide range of exercises within the individual training program was seen as a decisive factor in finding the right arrangement for themselves. Conversely, the need to create their own exercise program could also be perceived as a laborious process and a level of independence they did not find desirable.

The big advantage is that you get the videos in the Safe Step program, but it's difficult when you have to sit and watch them all to be able to choose.

Man 1; individual interview

#### Encourage the Person to Experiment and Self-initiate the Behavior (MBCT 7)

The subcategories “A comfort zone for action” and “Seizing the opportunities” capture expressions of how participants implemented the strategies and proposals given in the program and found ways to do the exercises that worked with their routines and motivation.

Comfort zones for performing the exercise were developed from suggestions in the program and described as enjoyment experimenting or stick to a structured training routine. Some participants found experimentation to be an enjoyable aspect, for example, by performing the exercises in outdoor environments and integrating them into their everyday routines. Others expressed their preference for the comfort of a familiar routine, and the commitment to complete the requested exercise amount could be perceived as a relatively high commitment. Another aspect of experimentation with the program, beyond the suggestions given to the participants in the program, was spontaneous attention given to capturing opportunities for exercise, both in terms of time and place for practice. One conversation was as follows (focus group interview 2):

Man 1: I really cracked the code of doing extra exercises.

Woman 1: With the toes?

Man 1: No, all kinds of situations. When I walk around the apartment, it suddenly hits me, and I start walking on my heels.

The ability to seize opportunities was described as something that was learned and evolved over time.

Taken as a whole, the autonomy-based categories highlight the importance and desire for opportunities for choice and personalization when using the digital exercise program.

### Competence Supportive Techniques

The categories created from the data analysis demonstrated that competence was seen as something that was developed through supported confidence and a strengthened inner locus of causality. Steps were taken to tailor the program to individual needs, rationales, and life circumstances, thus contributing to the “satisfaction” of the need for competence.

#### Assist in Setting an Optimal Challenge (MBCT 17)

The participants experienced support to set a suitable challenge, and the confidence and prerequisites for doing so were represented in the subcategory “Navigated by confidence.”

The digital exercise program was expressed as support for implementation of new exercise routines and assisted in setting weekly exercise goals from suggestions provided by the program. Variations in confidence in the participants’ own ability to achieve an optimal challenge were noted. For those with greater confidence, the suggestions provided in the exercise videos were enough to give a sense of capability to find a suitable exercise intensity and enabled them to adapt quickly, for example, to pain, by switching to easier or more difficult exercises.

I have always adapted to what suits me best because of my pain.

woman; focus group interview 2

Some expressed confidence in progressing their exercise but felt restricted due to limited progression options for their capability. Self-managing exercise progression could also be experienced as a challenge due to fear of having difficulties with the technology, voiced as follows:

I haven’t tried to change exercises because I worried there would be trouble with the equipment. Maybe it would not work.

Man 2; individual interview

#### Offer Constructive, Clear, and Relevant Feedback (MBCT 18)

The importance of nuanced and personalized feedback to support reflection on training performance was emphasized in the subcategory “Support for own reflection.”

The in-application statistics were communicated as clear and supportive for own reflections. Several benefits of being able to track changes over time were presented, and they were voiced as follows:

You get a grip of the situation.

Man 3; questionnaire

Statistics are always revealing.

Man 1; questionnaire

To be able to convey causes to adaptions through notes was expressed as a basis for being able to do a better self-evaluation of the training period. Further, the content of the feedback messages sent by the virtual physical therapist could either be perceived as supportive and a trigger for reflection, or lacking meaningful tailored feedback.

I like to go back and see which exercise I did not do when it [the virtual physical therapist] says I did not do them all. But, they appear when I have already done my training and I am not really interested to read much then.

Man; focus group interview 1

There was thus both “satisfaction” and “frustration” regarding support for own reflections.

#### Help Develop a Clear and Concrete Plan of Action (MBCT 19)

The subcategories “Initiation through reminders” and “Keep the exercise in mind” demonstrated feelings of support to develop new training routines.

Receiving reminders to support the initiation of the training generated diverse opinions. It was expressed that to do the exercise, the thought of exercise needed to be kindled, and for some, the reminders served that purpose. However, the lack of choices regarding when to receive them and how often was perceived as frustrating. By this, the need for tailored options was stressed. One conversation was as follows (focus group interview 2):

Woman 1: I would still like to be able to receive reminders.

Woman 2: Yes, preferably a few, but not too many. Just enough would be the best.

The weekly planning was highlighted as providing a valuable structure and a learning instrument to develop individual exercise routines. The importance of flexible planning was strongly emphasized as follows:

Weekly planning should guide training and not be considered the law

Man; focus group interview 1

The plan is a goal, but reality controls when exercise is suitable.

Man 1; questionnaire

#### Promote Self-monitoring (MBCT 20)

The subcategories “Self-monitoring on own terms,” “Inspiring statistics,” and “Confirmation through bodily feedback” represented that self-monitoring was governed by personal preferences, individual needs, and bodily knowledge.

Self-monitoring of training was expressed to be on own terms. Overall, registering exercise was expressed to work well and became part of the workout routine. However, after a while, some of the participants chose to stop registering or created their own systems for taking notes of performance. Nonetheless, it was emphasized that registration supported development of routines.

Even though I appreciate to register my exercise, I exercise for my own sake, not anyone else’s, or the registrations.

Woman 6; individual interview

The in-application statistics were also expressed to encourage self-monitoring of performance. Informative overviews helped guide exercise management and acted as motivators to continue to follow progress. As encouraged in the exercise videos, bodily feedback informed decisions about the individual exercise program. Experiences of improvements or declines in physical status, such as fatigue and improved or declined strength or steadiness, augmented self-monitoring and contributed with guidance in the adaptations of exercise intensity, voiced as follows:

I felt really insecure when I was walking in the city center. Now when I am out walking I can actually look at my surroundings, which are actually really pretty [laughs].

Woman 6; individual interview

#### Support Confidence in Performance (Additional MBCT)

The subcategories “A supportive structure,” “Minimizing uncertainty,” and “Embodied integration” referred to feelings of ease of use of the program and developed confidence in performing the exercise.

To be able to initiate a new exercise routine, the importance of a supportive structure was stressed for easily getting started, as was ease of use of the digital program, stated as follows:

You should not be able to misinterpret anything.

Woman 5; individual interview

These important qualities translated to the experience of the Safe Step program. Nonetheless, needs were expressed of a more extensive introduction on how to manage the program. Key elements of minimizing uncertainty regarding how to perform the movements were being able to see the exercises in video format and simultaneously being able to hear the instructions, which were also seen as access to ongoing support.

I think video tutorials is much better than a picture with text. Moving instructions along with telling me how it is performed, you can't misunderstand that.

Man 4; individual interview

The clarity of the instructions was mentioned as a basis for gradual integration and transition from needing the support of videos to be able to perform the exercises, to continuing without support. The exercise repetitions created an embodied knowledge and integration, which could be experienced as follows:

If you have done the exercises for a long time, they are in your head. Now when I do them without the tablet I almost hear them speak.

Woman; focus group interview 2

Through a comprehensive understanding of the program, they were able to easily integrate relevant parts of the program at any given situation and thereby find new situations for practice.

Taken together, competence to self-manage exercise was supported by the clear structure of the digital program and guided by confidence to manage the program.

### Relatedness Supportive Techniques

The categories show the diversity of the participants’ feelings and needs for social support while exercising with the digital program. In this sense, the basic psychological need of relatedness was both “satisfied” and “frustrated.” Aspects both in the close use of the program and by age-related perceptions of exercise influenced experiences of relatedness.

#### Acknowledge and Respect Perspectives and Feelings (MBCT 8)

Emotions that arose while interacting with the environment during performance of the training is described in the subcategory “Inhibited by social norm.”

As expressed by the participants, choices made in the program were influenced by a need to comply with the expected social norm of how to perform physical activity. The need to conform to such a norm became a contributing factor to which exercises were selected, and influenced where the training felt comfortable to perform.

Jumping steps felt a bit silly to do around the apartment. It is quite close between the apartment buildings and you can see the room quite clearly from the other building (laughter).

Man 5; individual interview

Performing the exercises outdoors could be experienced as uncomfortable at first, but individuals could gradually grow accustomed to it.

#### Show Unconditional Regard (MBCT 10)

The subcategory “Connection to the messages” portrayed thoughts of the support given by the feedback messages.

The information received from the virtual physical therapist was either perceived as an encouraging influence or an unimportant element. The encouraging influence was substantiated by an appreciated pleasant and praiseworthy tone of the messages. It was valued that someone took an interest in their performance, and the messages were interpreted as nonautomatically generated.

It felt like someone really cared about that I participated.

Man; focus group interview 2

By others, the messages could be experienced as an unimportant element due to a feeling of impersonal content, expressed as follows:

Receiving the messages just became part of the routine.

Man; focus group interview 2

No interest was sparked, and as a consequence, the messages were read at the beginning, but gradually the interest was lost.

#### Prompt Identification and Seek Available Social Support (MBCT 14)

The subcategories “The two-sided social support” and “Sources for social support” summarized different needs and solutions for social cohesion and social support to facilitate a changed exercise behavior.

The interviews revealed the two-sided nature of the need for social support. On the one hand, a greater freedom of choice was expressed when self-managing exercise at home, which could also be time-saving, and voiced as follows:

I have had to be on time my whole life. I don’t want to do that anymore.

Woman 2; individual interview

On the other hand, some missed the social support and felt that a group context would be more enjoyable and help increase the frequency and intensity of the exercise. Exercising at home was communicated as requiring a particularly large commitment.

Too easily it happens that the one sitting on your shoulder says: not today, and not tomorrow either, so the training does not happen.

Woman 1; individual interview

Finding own ways to increase social support during training and to identify sources of support was described. The program could act as a tool for social interaction as participants sometimes shared their knowledge or invited others, in whom they had identified a need for exercise, to exercise with them.

When I visit my friends, I have almost overwhelmed them when I surprise them with: “Now, let's do some balance exercises”, and they say: “Have you gone crazy?” But, then we perform them, and they are no better than me. It has become a fun event.

Woman 2; individual interview

Several sources of social connection were addressed within the application. The older adults demonstrating the exercises in the exercise videos had for some become virtual “friends” to keep them company during exercise, and the voice giving instructions in the videos was also perceived as a pleasant company. The virtual physical therapist was spoken of in terms of him or her, but the content of the messages was perceived as the most important element and not the avatar.

In summary, the above categories make it clear that even with a fully self-managed digital program, ways to increase relatedness were found, both interpersonally and through the digital program. A desire for additional alternatives for support and social cohesion was communicated, as was an awareness and adaptation to social norms.

## Discussion

### Principal Findings

This study explored expressions of self-determination among older adults using a self-managed digital exercise program with behavior change support (the Safe Step program). The results demonstrate that the Safe Step program was experienced as providing a supportive structure and an opportunity to tailor the individual exercise program, which was appreciated, although the freedom of choice was sometimes experienced as challenging. Support for all three basic psychological needs when using the Safe Step program was expressed, but autonomy and competence were more central to the participants’ experiences than relatedness, and as such, they were identified as primarily “satisfied.” The basic psychological need of relatedness was appreciated when present, but was requested by some participants, and therefore, it was identified as somewhat “frustrated.”

Fundamental to the participants’ expressions of autonomy was the experienced freedom of choice when using the digital program, as well as the possibility to adapt the exercise routines to their personal preferences and circumstances. Offering the opportunity to make informed and reflective choices is considered supportive for autonomy [16] and strengthening for behavioral engagement [29]. In accordance with our findings, a qualitative study involving older adults and health care professionals suggested that digital technology should enable participant independence and self-control in changing physical activity behavior and additionally should be perceived as nondemanding [30]. Further, older adults’ rationales, preferences, and possibilities to influence their fall prevention exercises have been found to be important to support a sustained intervention engagement [31].

When using digital technology, intervention engagement has been suggested to be affected by need satisfaction in different spheres as follows: adoption of new technology; interaction with the interface; engagement with a technology-enabled task; technology-supported behavior; link between technology and overall well-being; and societal impact [7]. As such, autonomy is experienced not only in the spheres of the interface or task through ease of use and choices, but also by extension through increased volition, removal of obstacles, or augmentation of capabilities in matters of daily life [7]. Increased autonomy in daily life was portrayed as an intervention effect in our study through increased confidence to experiment with the training and better capabilities to perform everyday tasks challenging balance and strength.

Support for competence to self-manage exercise was expressed as developed from a clear structure and clarified expectations, although a sustained lack of confidence to manage the digital program was perceived by some. Together with older adults, we developed techniques supportive for self-management of exercise. As presented in the competence-based categories, they were overall perceived as supportive for developing personalized exercise routines and confidence in performing them. Taken together, the Safe Step program supported a sense of capability, increasing the sense of self-efficacy for exercises, which was also found in a previous qualitative study [11]. In contrast, in other studies, behavior change techniques associated with self-regulation have been found less supportive for exercise self-efficacy among older adults [15]. However, the more positive attitude presented in our study could be related to older adults’ awareness of the self-regulatory nature of the intervention at enrollment.

In this study, the visual guidance in the exercise videos was highlighted as especially supportive for feeling confident in exercise performance. In concurrence with our results, demonstration of exercises has previously been suggested as helpful in supporting older adults’ changed physical activity behavior [15,30]. Moreover, in the Behavior Change Technique Taxonomy v1 (BCTTv1) [32] “Demonstration of the behavior” and “Instructions on how to perform the behavior” are specific behavior change techniques. In a systematic review and meta-analysis of SDT strategies used in health interventions, these techniques have been suggested to relate to the strategy of structure. Structure was defined as follows: “practitioners set parameters within which choice and agency can take place and provide support to initiate action” [19]. Structure has been suggested to be both autonomy [19] and competence supportive [33]. This overlap is, however, not uncommon as strategies often can be supportive for more than one need [16]. In the classification of MBCT, we found no support for classifying expressions related to how the program provided structure and supported competence in the actual performance of the exercise. Hence, an additional competence-based category was created as the experiences expressed by the participants were related to increased confidence in performing the activity and support for improving skills.

A wish for increased relatedness in connection to training or a belief of additional benefits of group-based exercise was expressed in this study. This preference for group training has also been expressed in previous studies as a way to make the exercise delivered by digital technology more enjoyable [30]. A way to support relatedness in digitally supported home-based training could be to include tips on how the exercises can be performed with others. Interestingly, some participants had already started to use the application in their interaction with others, indicating both a pride in their efforts and a confidence in their capability to guide others. Apart from using the program as a facilitator of relatedness, connectedness to the digital program itself was conveyed in the interviews. Similar results presented in previous research regarding relatedness and telecare suggest that the concept of relatedness should be broadened beyond the interpersonal context to incorporate organizational and technical concerns in order to provide a more nuanced view [34]. It is worth noting that the importance of relatedness has previously shown inconsistencies in training contexts, which may be partly due to variations in the design and measurements of the studies [35,36]. Still, as also mentioned in this study, individuals might prefer to undertake fall prevention exercise in solitude [37], which may reduce the importance of supporting relatedness in that activity in contrast to autonomy and competence [18].

We found that participants sometimes avoided certain public settings or exercises due to an awareness of sociocultural ageist norms for exercise and the aging body [38]. Previous qualitative results illustrated that negative age perceptions for physical activity can reside within the individual and must be overcome to facilitate sustained exercise behavior. Furthermore, older adults felt that they were treated differently because of their age when exercising in public spaces or social situations [39]. As part of counteracting such ageist norms, the exercise videos in the Safe Step program were filmed with older adults. However, our results indicate that further efforts could be made. One development could be to incorporate more films with other participants who voice emotions and problematize or even challenge restricting age norms.

Satisfaction of the basic psychological needs has previously been found to support exercise behavior among older adults. However, in likeness with our results, the representativeness of the different needs varied [35,40]. Results from a meta-analysis showed that no individual SDT-based technique to promote motivation for health behavior change was predominant, and a need supportive environment should contain multiple techniques to promote health behavior change [19]. These results are comparable to the results of our study, as the participants’ experiences were more shaped by the sum of behavior change support than by the parts.

### Theoretical and Methodological Discussion

In our previous research where older adults’ exercise preferences and motivators in the context of fall prevention were explored, it emerged that SDT captured the essence of the findings well [41]. Accordingly, during the developmental phase of Safe Step, the SDT did influence the content and layout, but the theory was not explicitly used to frame the studies or the interview guides. Because of the theory’s influence, the classification of MBCTs [20] based on the SDT was used as an analytical matrix in the deductive stage of the qualitative content analysis in this study. In 2019, Gillison et al published a meta-analysis of SDT-based techniques to support health behavior change [19]. This meta-analysis presents an overview of techniques used in SDT interventions and could have been used as an analytical matrix. However, we found the techniques in the classification of MBCTs to be more comprehensively described and therefore easier to use in the analysis, with the additional advantage of being derived from an expert consensus.

In our study, participants expressed support for behavior change in the Safe Step program related to 11 of 21 MBCTs. However, unrepresented MBCTs may still be present in the program but were not specifically asked about in the data collection and were therefore not expressed (eg, the noncontrolling and positive language used in the messages sent by the virtual physical therapist). The SDT has been found applicable to the context of physical activity [35], older adult exercise behaviors [40], and eHealth [42]. However, in relation to our fully self-managed digital context, we encountered a few difficulties when using the classification system. To overcome this, we had to interpret some MBCTs to the fully self-managed and digital context of our study, as many of the MBCTs are facilitated by the involvement of a health care professional or social agent. In order to use the classification of MBCTs to enable better comparisons among self-determination–based interventions in the health domain, especially in this digital era, we suggest that the definitions of the MBCTs should be broadened and perhaps also clarified. For example, the ability to see the exercises in video format and the ease of use of the digital exercise program were in our study stated as important to build confidence in performance and support independence. We were unable to classify these techniques used to support actual performance and increase confidence in performing health-related behaviors. Of note, the analysis contributed with a proposal for an additional competence-based MBCT addressing supportive structures for the performance of behavior.

Methodological trustworthiness has been strived for in various ways in the execution and writing of this study [22]. Three data collection methods were used, including self-reported questionnaires, and individual and focus group interviews. The triangulation of data collection methods enabled looking at data from different perspectives. With the addition of individual interviews and comments in the questionnaires, all participants using the Safe Step program in the feasibility study were represented in the data collection. Overall, older adults with different ages, technology experiences, and exercise adherences were represented in both the individual and focus group interviews to capture the diversity of experiences [43]. Unfortunately, in this study, group compositions regarding women and men became uneven in the focus group interviews, which may have influenced the responses. Moreover, we recognize the fact that it would have been valuable to explore the views of participants who discontinued the intervention in order to explore possible frustration of the basic psychological needs. Furthermore, the experiences of those conducting the interviews varied, which resulted in both novice and in-depth follow-up questions. The analysis was continuously discussed and triangulated by the authors who had different methodological and theoretical backgrounds, professional experiences, or insider and outsider perspectives, which strengthened the credibility of the results. To enable assessment of transferability to other study contexts and target groups/populations, we have described the participants, research context, and methods of the study thoroughly, according to recommendations on how to improve transferability in qualitative studies [23]. We have also strived for a rich description of participants’ experiences, with exemplifying quotes.

### Conclusions

This study demonstrated that older adults using a fully self-managed digital exercise program for fall prevention (the Safe Step program) expressed support for all three basic psychological needs, though autonomy and competence were predominantly supported compared with relatedness. The Safe Step program supported the development of new exercise routines, and the program was found adaptable to one’s own capacities and objectives, and therefore fostered feelings of engagement and ownership in the self-management of exercise. Self-managing fall prevention exercise was found to entail both feelings of freedom and restriction. By using the classification system for MBCTs as an analytical matrix, suggestions for further development of the classification system were made to better suit more digital health interventions. This study proposes to add an MBCT to the classification system that captures support to strengthen a person’s competence in performing an activity or task.

## Appendix

Multimedia Appendix 1 Overview of the Safe Step project according to the complex intervention framework.

Multimedia Appendix 2 Overview of the areas of questions in the different data collections.

## Abbreviations

MBCT: motivation and behavior change technique
SDT: self-determination theory
