# Supplementary figures and images for: Older Adults’ Experiences of Behavior Change Support in a Digital Fall Prevention Exercise Program: Qualitative Study Framed by the Self-determination Theory
Source: J Med Internet Res. 2021 Jul 30;23(7):e26235. doi: 10.2196/26235 (PMC8367180; doi:10.2196/26235)

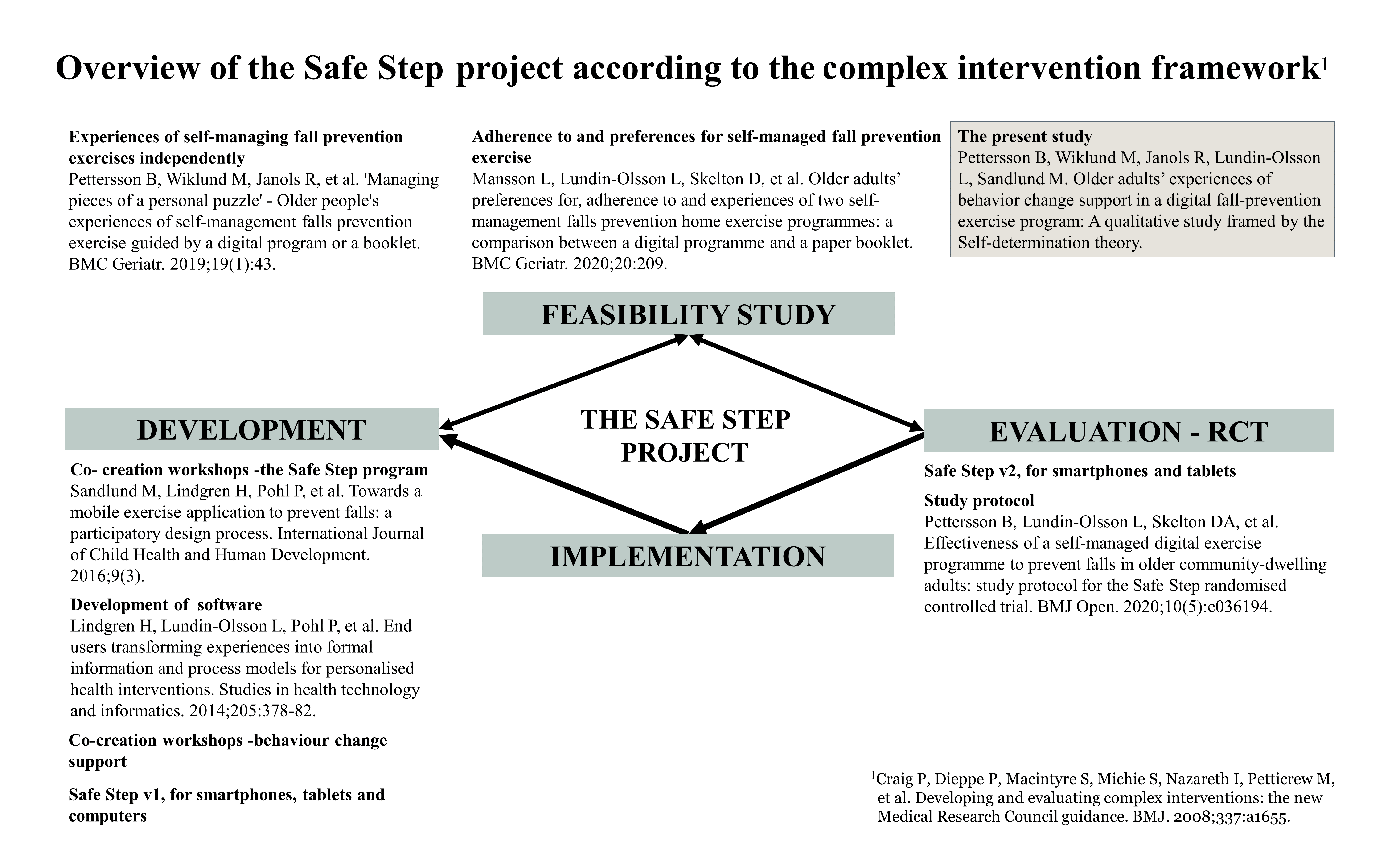

Supplement: Multimedia Appendix 1 [file jmir_v23i7e26235_app1.png]

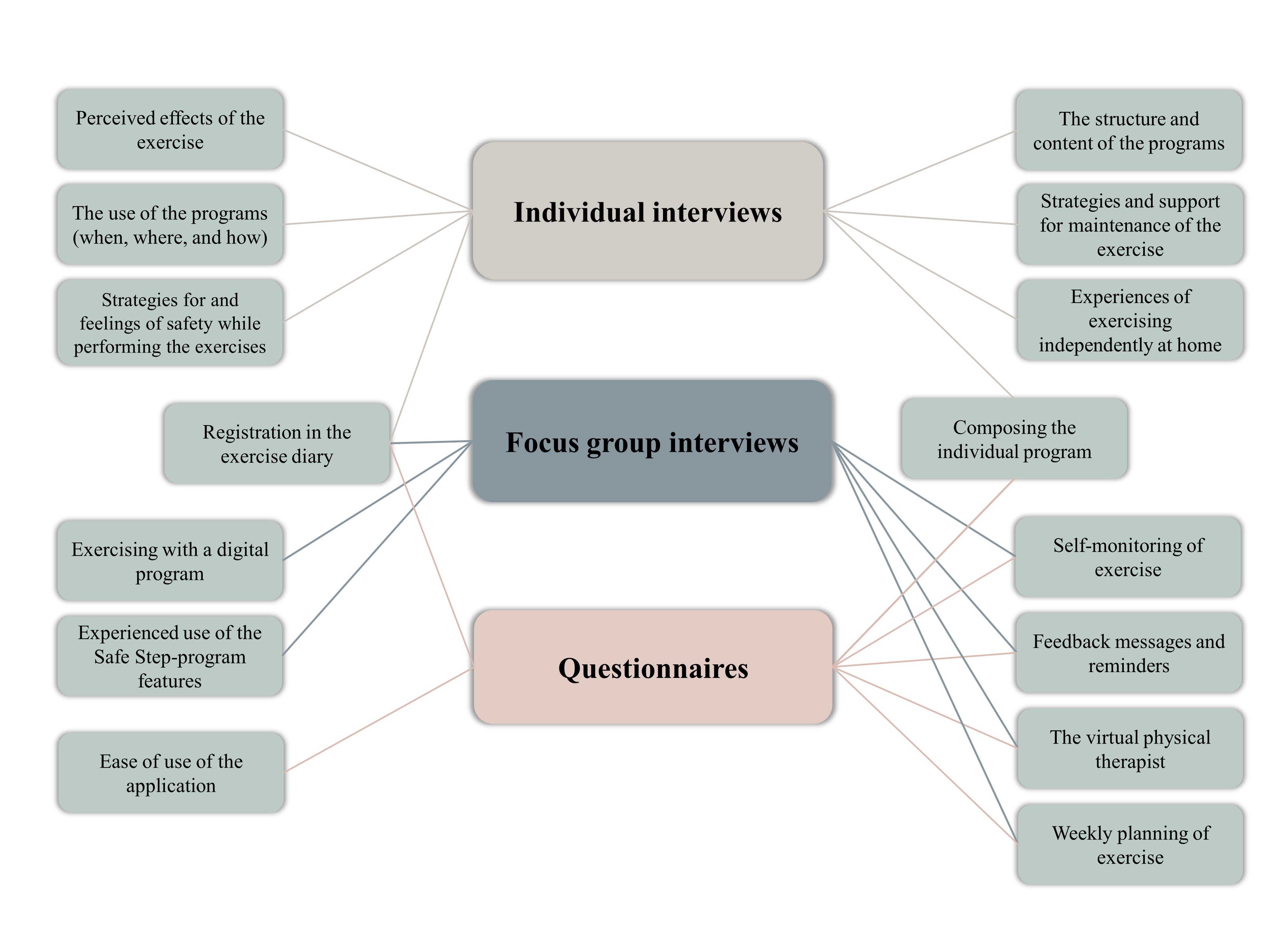

Supplement: Multimedia Appendix 2 [file jmir_v23i7e26235_app2.png]
